# Bilateral Pulmonary Embolism in a Hospitalized Ulcerative Colitis Patient

**DOI:** 10.7759/cureus.21861

**Published:** 2022-02-03

**Authors:** Karen Medgyesy, Jamie Horrigan, Micheal Tadros

**Affiliations:** 1 Gastroenterology and Hepatology, Albany Medical Center, Albany, USA; 2 Internal Medicine, Dartmouth-Hitchcock Medical Center, Lebanon, USA

**Keywords:** pulmonary embolism (pe), crohn’s disease (cd), ulcerative colitis (uc), venous thromboembolism prophylaxis, inflammatory bowel disease

## Abstract

Patients with inflammatory bowel disease (IBD) are at higher risk of venous thromboembolism (VTE), though physicians may be unaware of this risk or hesitant to start pharmacologic VTE prophylaxis in the presence of active gastrointestinal bleeding. We report a case of a 38-year-old patient hospitalized with acute severe ulcerative colitis (UC) who was not placed on pharmacologic VTE prophylaxis and developed bilateral pulmonary embolism (PE). The patient's UC did not respond to medical therapy. Due to his PE, the patient’s total proctocolectomy was delayed six months. He also developed a large pelvic hematoma after colectomy requiring further surgical intervention. Hospitalized inflammatory bowel disease (IBD) patients require pharmacologic VTE prophylaxis unless they have life-threatening bleeding.

## Introduction

Inflammatory bowel disease (IBD) is a chronic inflammatory condition of the gastrointestinal (GI) tract that includes Crohn’s disease and ulcerative colitis (UC). The prevalence of IBD has increased from 3.7 million affected individuals in 1990 to 6.8 million affected individuals in 2017 worldwide [1]. Both conditions cause relapsing and remitting episodes of abdominal pain, diarrhea, and weight loss [2]. Extra-intestinal symptoms of the joints, skin, hepatobiliary tract and eye occur in IBD as well, some of which correlate with disease activity [3].

Additionally, patients with IBD are at a two-to-three-fold increased risk of developing venous thromboembolism (VTE) compared to the general population, which encompasses deep vein thrombosis and pulmonary embolism (PE) [4]. This risk increases with hospitalization and active disease [5]. The etiology of this hypercoagulable state is multifactorial and incompletely understood. Molecular factors, such as upregulation of coagulation factors and increased platelet count due to systemic inflammation, and clinical factors, including use of certain medications (corticosteroids, tofacitinib) and surgeries related to IBD, are thought to play a role in VTE development [4].

Current guidelines support that all hospitalized IBD patients with non-severe GI bleeding should receive pharmacologic VTE prophylaxis with low molecular weight heparin, low-dose unfractionated heparin, or fondaparinux [6]. We present a case of PE in a hospitalized UC patient, causing complications and delay in a necessary colectomy.

This article was previously presented as a meeting abstract at the 2020 Hospital Medicine Virtual Competition held by the Society of Hospital Medicine in August 2020.

## Case presentation

**Figure 1:**
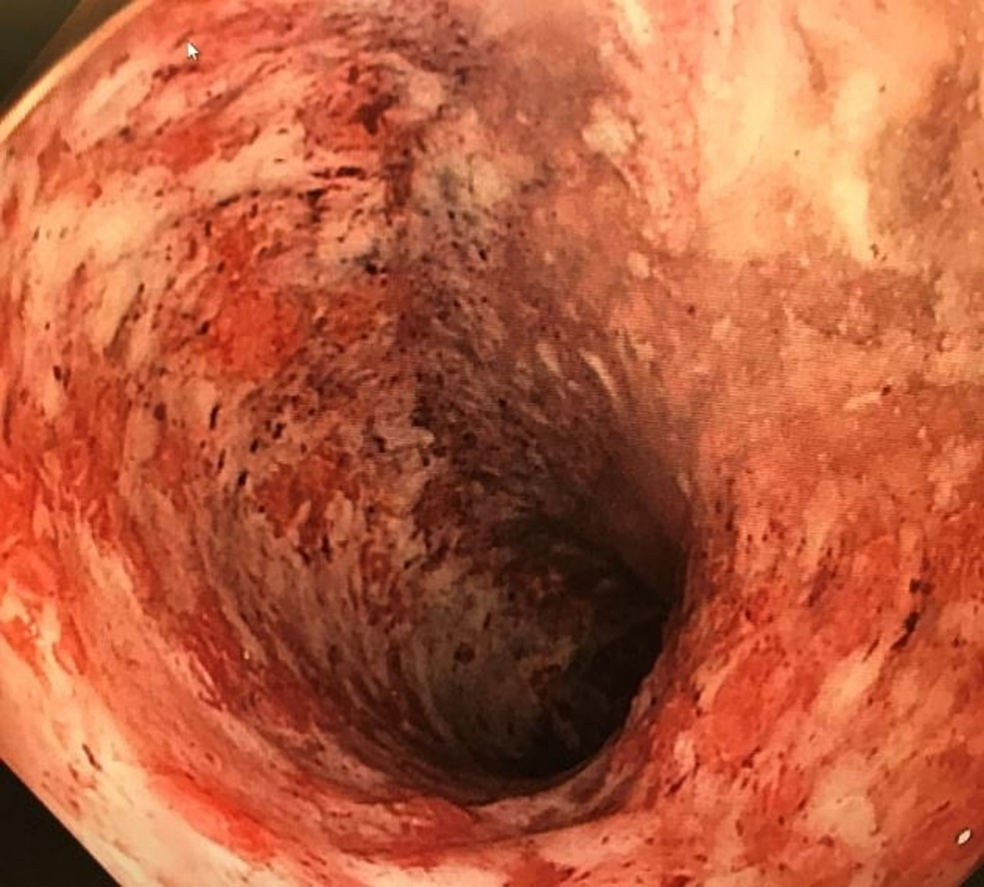
Severe diffuse pancolitis (Mayo Score 3) with pseudomembrane in the descending colon.

A 38-year-old male with a five-year history of UC was hospitalized with an acute severe UC flare after experiencing up to 42 bloody bowel movements daily and a 60-pound weight loss over the past year. The patient was found to have fulminant ulcerative pancolitis with pseudomembranes (Figure 1). Clostridioides difficile testing was negative. His disease was refractory to maintenance therapy with high dose prednisone (60 mg), 10 mg/kg of infliximab every six weeks, and 75 mg of 6-mercaptopurine.

The patient was admitted for intravenous corticosteroids, cyclosporine salvage therapy, and total parenteral nutrition with plans for colectomy if his disease did not respond to therapy. Due to the patient’s hematochezia, he was given sequential compression devices for VTE prophylaxis. Despite his hematochezia, his hemoglobin remained stable, and he did not require packed red blood cell transfusion.

**Figure 2:**
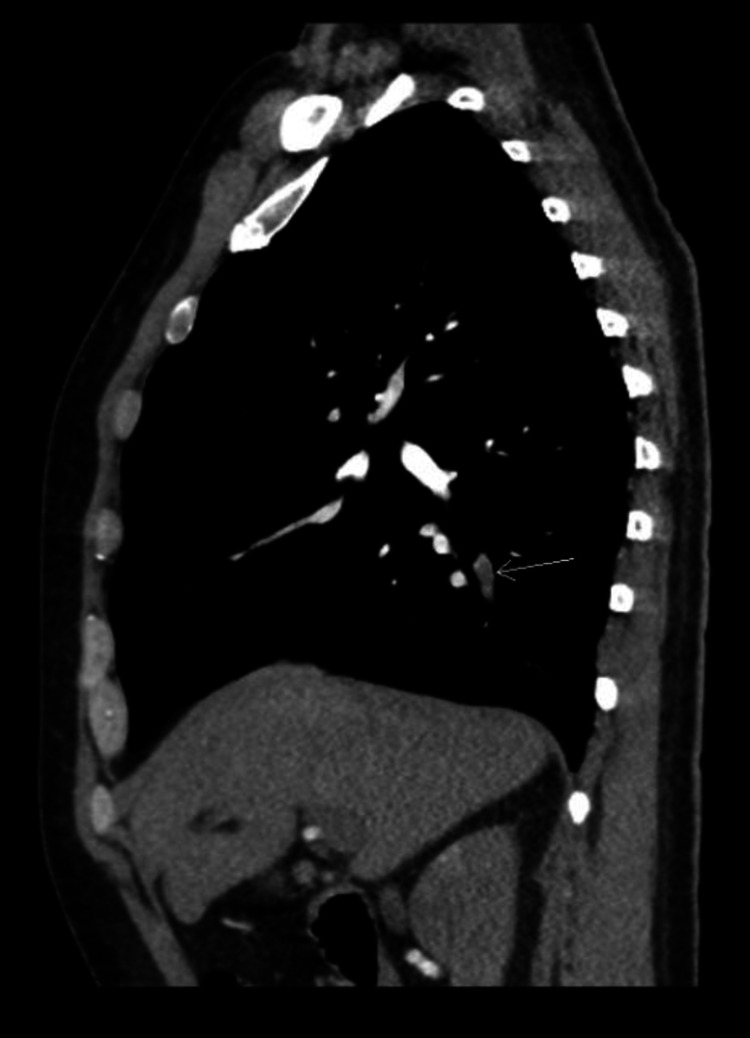
Sagittal view of the contrast-enhanced computed tomography scan of the chest demonstrating pulmonary embolism in the right lower lobe (arrow).

On hospital day three, the patient reported chest pain and shortness of breath. Contrast-enhanced computed tomography revealed pulmonary emboli involving segmental arterial branches to the right and left upper lobes and the right lower lobe (Figure 2).

Following his PE diagnosis, the patient was started on a heparin drip and, interestingly, reported a decrease in the number of total bowel movements after starting anticoagulation. The patient was discharged on warfarin for anticoagulation and prednisone with plans to start tofacitinib as an outpatient. The patient, unfortunately, did not respond to treatment with tofacitinib and 20mg prednisone as an outpatient and required a total proctocolectomy. However, due to surgical risks with his acute PE, his surgery was delayed six months from initial hospitalization. During the six-month waiting period, he remained on tofacitinib and prednisone as maintenance therapy. The patient had total proctocolectomy completed, but postoperative was complicated by a large pelvic hematoma requiring further surgical intervention. He was discharged and recovered well afterward.

## Discussion

Although literature demonstrates the increased risk of VTE in IBD patients, thromboprophylaxis in hospitalized patients with IBD needs to be better addressed. A single-center study of 3758 IBD admissions found that VTE prophylaxis is underutilized in 50% of IBD patients that developed VTE [7]. Another retrospective cohort study found that only 68% of hospitalized UC patients were prescribed VTE prophylaxis [8].

A more recent study of hospitalized patients between January 2013 and August 2018 found that IBD patients were less likely to be placed on VTE prophylaxis, with 79% of IBD patients placed on VTE prophylaxis, compared to 87% of hospitalized patients without IBD [9]. Additionally, IBD patients with the active disease under the age of 35 and those with hemoccult positive or frank blood on the digital rectal exam (DRE) are less likely to receive proper VTE prophylaxis [10]. This is unfortunate as an active disease state increases the risk of VTE development [4].

Underutilization of pharmacologic VTE prophylaxis in patients with IBD may be related to a lack of awareness as well as safety concerns regarding bleeding risk. In a survey of 591 gastroenterologists, 19.4% failed to acknowledge the increased risk of thrombosis in IBD patients, while 29.1% were unaware of practice guidelines for VTE prophylaxis in hospitalized IBD patients [11]. Another study suggested low VTE prophylaxis rates may be related to decisions made by physicians in departments lacking awareness of this topic [12]. The safety of anticoagulation in IBD patients is a concern as well. Hematochezia was also associated with decreased pharmacologic VTE prophylaxis despite no evidence of increased bleeding risk or transfusion requirements [9]. Other studies have shown no increase in the incidence of major bleeding in patients with IBD who received pharmacologic VTE prophylaxis [13,14].

Guidelines provided by the Canadian Association of Gastroenterology recommend anticoagulant prophylaxis for all hospitalized patients with IBD with low molecular weight heparin (LMWH), low-dose unfractionated heparin, or fondaparinux unless severe bleeding is present. Patients admitted for reasons other than an IBD flare, including those in clinical remission, and patients who have undergone major abdominal-pelvic or general surgery are included in this recommendation. For hospitalized IBD patients with severe IBD-related gastrointestinal (GI) bleeding, mechanical thromboprophylaxis, preferably with intermittent pneumatic compression (IPC), is recommended. Once the bleeding becomes non-severe, anticoagulant thromboprophylaxis is recommended during the hospital stay due to the increased effectiveness of anticoagulant prophylaxis in VTE prevention compared to IPC [6]. LMWH reduces the risk of thrombosis by 50% [15].

This case highlights the importance of VTE prophylaxis in IBD patients. These patients are at an increased risk for thromboembolic events with the potential for significant morbidity and mortality [16,17]. Physicians may be unaware of this risk or hesitate to initiate pharmacologic VTE prophylaxis in patients admitted with hematochezia due to increased bleeding risk. This runs contrary to studies that have shown no increase in major bleeding risk in patients with IBD who received pharmacologic VTE prophylaxis [9,13,14]. Anticoagulation should certainly be held in patients with life-threatening bleeding, and those patients should receive mechanical thromboprophylaxis, preferably IPC [6].

## Conclusions

This case illustrates the need for pharmacologic VTE prophylaxis in hospitalized IBD patients due to the increased risk of thromboembolic events. More awareness is needed among physicians that hospitalized IBD patients require proper pharmacologic VTE prophylaxis in the absence of life-threatening bleeding due to a higher risk of VTE as it carries the potential for significant morbidity and mortality in IBD patients. The benefits of pharmacologic VTE prophylaxis outweigh the risks.
